# Clinical features and management of atypical hemolytic uremic syndrome patient with *DGKE* gene variants: a case report

**DOI:** 10.3389/fped.2023.1162974

**Published:** 2023-06-29

**Authors:** Xiaomei Dai, Yu Ma, Qiang Lin, Hanyun Tang, Ruyue Chen, Yun Zhu, Yunyan Shen, Ningxun Cui, Zhongqin Hong, Yanhong Li, Xiaozhong Li

**Affiliations:** ^1^ Department of Nephrology and Immunology, Children’s Hospital of Soochow University, Suzhou, China; ^2^ Department of Respiratory Medicine, Children’s Hospital of Soochow University, Suzhou, China; ^3^ Institute of Pediatric Research, Children's Hospital of Soochow University, Suzhou, China

**Keywords:** atypical hemolytic uremic syndrome, DGKE gene, TMA, plasma therapy, chronic kidney disease (CKD)

## Abstract

**Background:**

Atypical hemolytic uremic syndrome (aHUS) with diacylglycerol kinase epsilon (*DGKE*) gene variant is a rare variant of thrombotic microangiopathy (TMA). The information on the clinical features, management and long-term outcomes of *DGKE*-aHUS patients have not yet been fully elucidated. The aim of this study was to report a novel variant of the *DGKE* gene in a Chinese population with aHUS.

**Case presentation:**

The present work reports a 7-month-old boy with aHUS, possibly triggered by gastrointestinal infection, without complement activation, with little response to plasma therapy and nephroprotective measures. The patient died during the 8th week of his hospital stay. The causes of death were intracranial hemorrhage and multiorgan dysfunction. Comprehensive WES of peripheral blood-derived DNA revealed two heterozygous variations in the *DGKE* exon region: NM_003647.2, c.610dup, p.Thr204Asnfs*4 and deletion of exons 4–6.

**Conclusions:**

This case suggest that atypical HUS with *DGKE* gene variant has a poor prognosis with a high mortality rate, which typically manifests in the first year of life and presents as a systemic disease with early-onset HUS with rapidly worsening renal function and chronic proteinuria. There is no specific treatment for *DGKE*-aHUS. There have an uncertain benefit of plasma therapy for *DGKE*-aHUS patients. The literature demonstrated that anti-complement therapy showed benefits for *DGKE*-aHUS with complement activation and autoantibodies during the overt TMA presentation but did not prevent TMA relapses. Early diagnosis and treatment may prevent complications and improve prognosis.

## 1. Introduction

*DGKE*-aHUS is an autosomal recessive disorder caused by “bi-allelic” loss-of-function variants in the *DGKE* gene that is characterized by microangiopathic hemolytic anemia, thrombocytopenia, and renal impairment (1–3). In recent years, the pathogenesis of aHUS has been found to be associated with genetic or autoimmune abnormalities leading to pathologic complement cascade activation (2, 4). Lemaire et al. (2) found that the recessive variant of the diacylglycerol kinase epsilon (*DGKE*) gene is an important pathogenesis of a novel aHUS. *DGKE* is found in the endothelium, platelets and podocytes and is a member of the lipid kinase family of proteins that show anticoagulant effects via diacylglycerol-mediated activation of protein kinase C (4). Previous studies have shown that *DGKE* variants can lead to podocyte damage, glomerular capillary microthrombosis, and vascular occlusion (5, 6). Lemaire and his colleagues (2) believed that *DGKE* genetic variation is an independent mechanism of the pathogenesis of aHUS, which theoretically does not affect the complement pathway. Sanchez CD et al. (7) suggested that abnormal complement and *DGKE* variants play an interactive role in the pathogenesis of aHUS. There are various phenotypes of disease associated with the mutated *DGKE* gene (8). To date, many cases of recessive variants in the *DGKE* gene that cause aHUS have been reported abroad. Reports of variants in the *DGKE* gene are still rare in China. Here, we present a case of a Chinese family with a proband diagnosed with aHUS in infancy and phenotypes of hypertension, gross hematuria and proteinuria. Whole-exome sequencing (WES) demonstrated a heterozygous insertion variant: c.610dup, p.Thr204Asnfs*4 and heterozygous deletion variation in exons 4–6 of this gene.

This research was reviewed and approved by the Ethics Committees of the Children's Hospital of Soochow University (Suzhou, China), and the study was performed in accordance with the Declaration of Helsinki. We collected peripheral blood samples from the proband and his parents after genetic counseling and obtained informed consent for molecular investigation.

## 2. Case presentation

### 2.1. Clinical presentation

A 7-month-old Chinese boy was referred to the pediatric nephrology and immunology department of our hospital with eyelid edema for more than 2 months and limb swelling for 1 week. In the medical history, there had been recurrent cough that continued for some days. He had a pale complexion, with periorbital and bilateral pitting pedal edema on clinical examination, but no jaundice, petechiae, purpura, or lymphadenopathy. There was no history of tachycardia or tachypnea. There was no hepatosplenomegaly. His blood pressure (81/62 mmHg) was in the normal range at admission. The child had a history of amniotic fluid contamination at birth and no history of asphyxia. Neither the boy nor his family had a significant history of conditions such as kidney-related diseases or autoimmune diseases. However, the grandmother died of hematological disease.

Investigations 3 days prior at clinics of another hospital showed a hemoglobin (Hb) level of 62 g/L without thrombocytopenia (platelet count 396 × 10^9^/L). Serum lactate dehydrogenase (LDH) was elevated (579 U/L) (normal <382 U/L). An elevated serum cholesterol of 5.38 mmol/L was noted at that time, along with range proteinuria (1+) and hypoalbuminemia (serum albumin 28.8 g/L). There was no gross or microscopic hematuria. The serum C3 and C4 levels were normal. The laboratory values with reference values are shown in the Table 1. The boy was immediately referred to our hospital with a referral diagnosis of “nephrotic syndrome (NS)”.

**Table 1:** Clinical characteristics of the aHUS patient with *DGKE* variant.

Characteristics	Laboratory value	Reference value	Unit
Hemoglobin	62	120–140	g/L
MCV	86	80–100	fL
Reticulocyte count	9.3	0.5–1.5	%
Schistocytes count	3	0	%
Platelet count	23	100–300	×10^9^/L
SCr	0.62	0.20–0.40	mg/dl
BUN	5.66	2.9–8.2	mmol/L
Cystine	1.57	0.59–1.03	mg/L
C3	0.89	0.7–1.4	g/L
C4	0.21	0.1–0.4	g/L
LDH	585.1	172–382	U/L
Hematuria	4,627.9	0–4.5	/µl
Proteinuria	+++	− or ±	–
PT	36.2	11–14	s
APTT	58.6	28–45	s
Fibrinogen	4.26	2–4	g/L

APTT, activated partial thromboplastin time; BUN, blood urea nitrogen; C3, serum complement C3 level; C4, serum complement C4 level; LDH, lactate dehydrogenase; MCV, mean corpuscular volume; PT, prothrombin time; SCr, serum creatinine.

**Figure 1:**
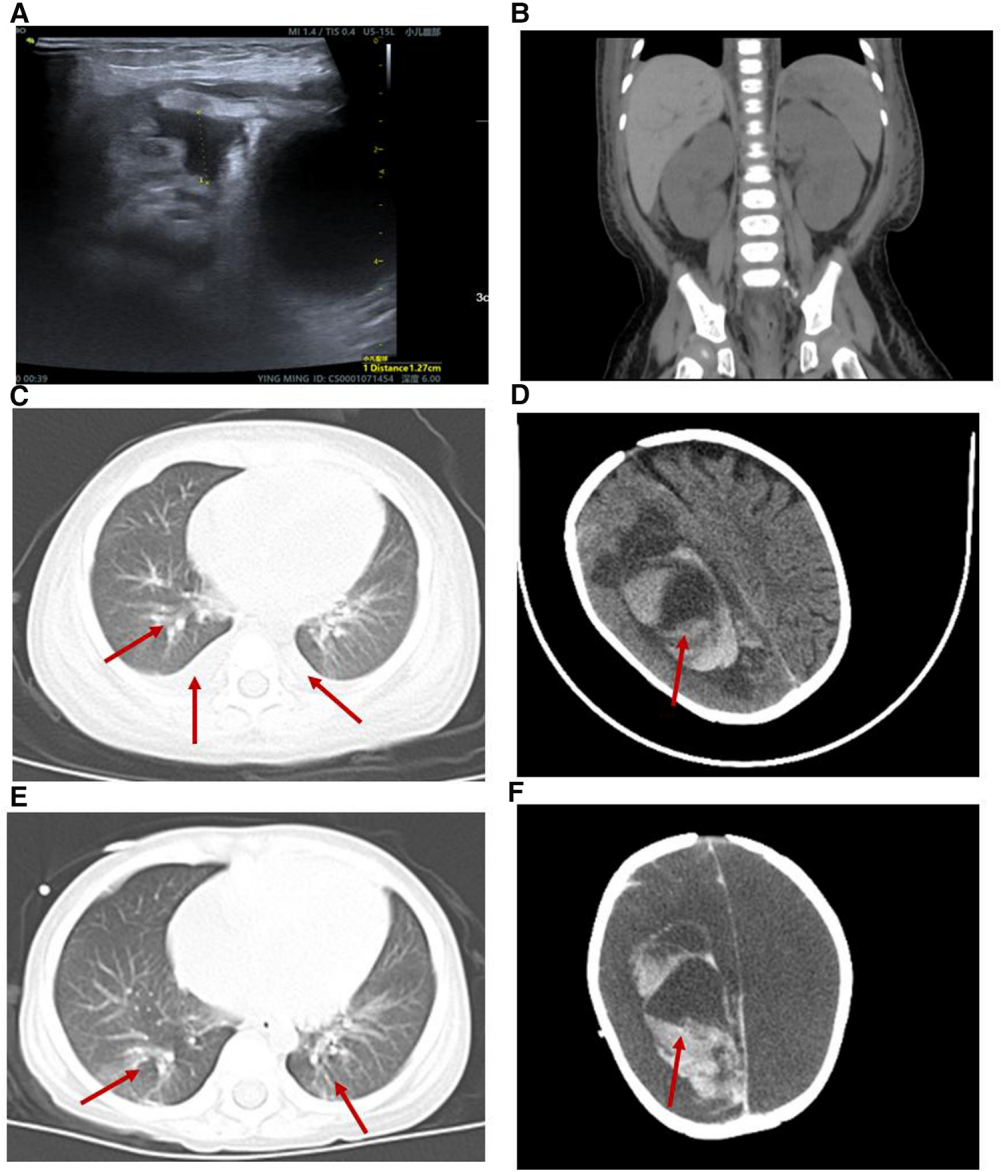
Radiological alterations during the patient's hospitalization. (**A**) B-ultrasound showed a small amount of abdominal effusion. (**B**) Abdominal computed tomography (CT) showed that the kidney, liver and spleen increased in volume with a low-density image. (**C,E**) Chest CT displayed both lung pneumonia and partial atelectasis of the upper lobe of the right lung. (**D**) Head CT showed that the subarachnoid hemorrhage during the 6th week of his hospital stay. (**F**) Head CT showed that the subarachnoid hemorrhage was more extensive during the 8th week of his hospital stay.

The patient presented with likely NS with anemia for five days after admission but no proteinuria or hypoalbuminemia. The direct Coombs test was negative. On hospital day 6, the patient presented with “hemolytic uremic syndrome (HUS)” following episodes of vomiting and diarrhea with fever. There were systemic pitting pedal edema, oliguria, petechiae, and soy sauce-colored urine at that time, along with hypertension (129/89 mmHg). Laboratory workup revealed normocytic hemolytic anemia with an Hb level of 62 g/L, mean corpuscular volume (MCV) of 86 fL, elevated reticulocyte count of 9.3%, schistocytes count of 3% on the peripheral smear, thrombocytopenia (platelet count 23 × 10^9^/L), a serum creatinine (SCr) level of 0.62 mg/dl, blood urea nitrogen (BUN) level of 5.66 mmol/L, cystine (C) level of 1.57 mg/L, C3 level of 0.89 g/L, C4 level of 0.21 g/L, a markedly increased lactate dehydrogenase (LDH) level of 585.1 U/L, hematuria (red blood cells 4,627.9/µl), and proteinuria (3+). Coagulation function, including prothrombin time (PT), activated partial thromboplastin time (APTT) and fibrinogen, was elevated (36.2 s, 58.6 s and 4.26 g/L, respectively). The serum bilirubin, AST, ALT, and serum electrolyte levels were within normal limits. The activity of a disintegrin and metalloproteinase with a thrombospondin type 1 motif, member 13 (ADAMTS13), was normal. The stool rotavirus test was positive. Stool culture grew *Streptococcus* and *Escherichia coli* (*E. coli*), and stool multiplex polymerase chain reaction (PCR) showed that *Shigella*, *Salmonella*, *E. coli* O157, and pathogenic *Vibrio* were not detected. The infectious workup was notable for a negative respiratory viral panel, negative special viral panel (human parvovirus B19, EB virus, cytomegalovirus, rubella virus, herpes simplex virus, hepatitis virus), negative *Mycobacterium tuberculosis*, and negative blood culture. Antinuclear antibodies were negative. The chest x-ray was negative. B-ultrasound showed a small amount of abdominal effusion (Figure 1A). Abdominal computed tomography (CT) showed that the kidney, liver and spleen increased in volume with a low-density image (Figure 1B). The echocardiogram showed a central atrial septal defect (2.3 mm) with a normal ejection fraction (EF 66%). The 24 h urine protein quantification was not performed due to the difficulty in collecting 24 h urine. Moreover, due to his uncontrolled hypertension and thrombocytopenia, the patient could not undergo a kidney biopsy.

Given the concern for HUS, broad-spectrum antibiotics and fresh frozen plasma infusions were given to treat his gastrointestinal infection and TMA. The following other treatments were employed: intravenous immunoglobulin (IVIg) infusions, human prothrombin complex infusions, albumin infusions (due to a decreased level of albumin), red blood cell infusions, platelet infusions, intramuscular injection of vitamin K1, low-dose methylprednisolone for anti-inflammatory treatment, antihypertensive treatment (nifedipine and metoprolol), and symptomatic treatment.

**Figure 2:**
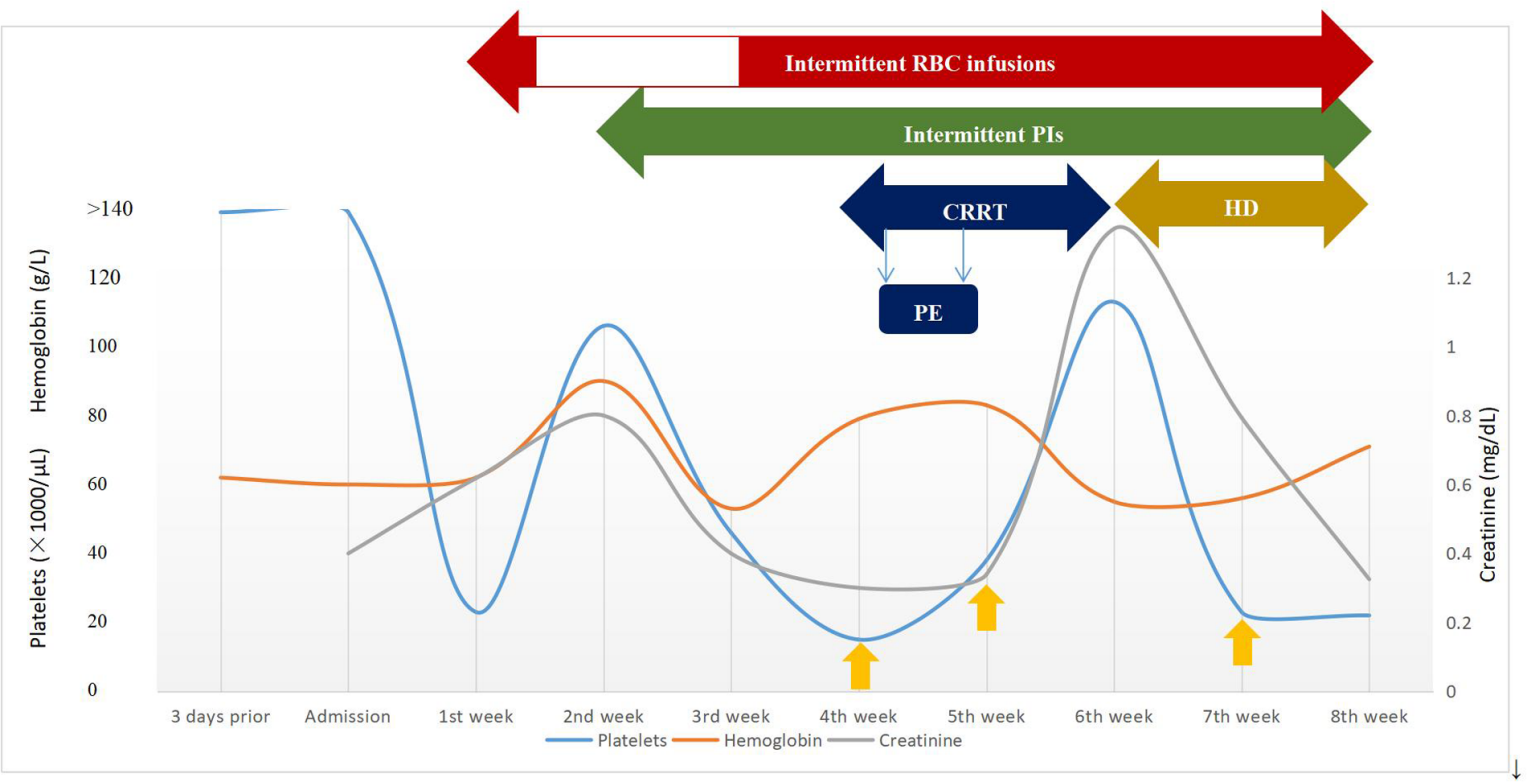
Timeline of clinical course and laboratory data during episodes of aHUS and management. The y-axis on the left shows platelets and hemoglobin levels and the y-axis on the right shows creatinine levels. The yellow upward arrow represents platelet infusion. CRRT, continuous renal replacement therapy; HD, hemodialysis; PE, plasma exchange; PIs, plasma infusions; RBC, red blood cell.

The patient was diagnosed further with aHUS, had been administered plasma infusions (PIs) discontinuously and had required plasma exchange (PE) 2 times during the PICU hospital stay. Given the trend of acute respiratory distress syndrome (ARDS) with worsening pneumonedema and pneumorrhagia, mechanical ventilation (MV) was given for 7 days. There was a transient remission of respiratory symptoms. However, given the trend of worsening renal function [elevated levels of blood urea nitrogen (BUN)], progressing edema, and worsening hypertension, continuous renal replacement therapy (CRRT) and hemodialysis (HD) on alternating days were given to treat his acute kidney injury (AKI) for 13 days and 15 days, respectively. The patient's renal function and hematologic parameters (stage 3 AKI with gross hematuria) worsened during the 6th–7th week of the hospital stay while HD and PI therapy was continued, as depicted in Figure 2. Subsequently, the patient developed a hypertensive emergency with features of intracranial hypertension leading to right temporoparietal hematoma and subarachnoid hemorrhage, as depicted in Figure 1D. He required multiple drugs (furosemide, nitrate, glycerin fructose) for the control of hypertension and intracranial hypertension. Ultimately, he still had a recurrence of ARDS and deteriorated to a stage of involuntary respiration requiring high ventilatory settings with pulmonary hemorrhage, gastrointestinal hemorrhage, and multiorgan dysfunction. Chest CT displayed both lung pneumonia and partial atelectasis of the upper lobe of the right lung (Figures 1C,E). Head CT showed that the subarachnoid hemorrhage was more extensive than before (Figure 1F). The patient's parents decided to withdraw the patient from further management. The patient died during the 8th week of his hospital stay.

### 2.2. Whole exome sequencing

**Figure 3:**
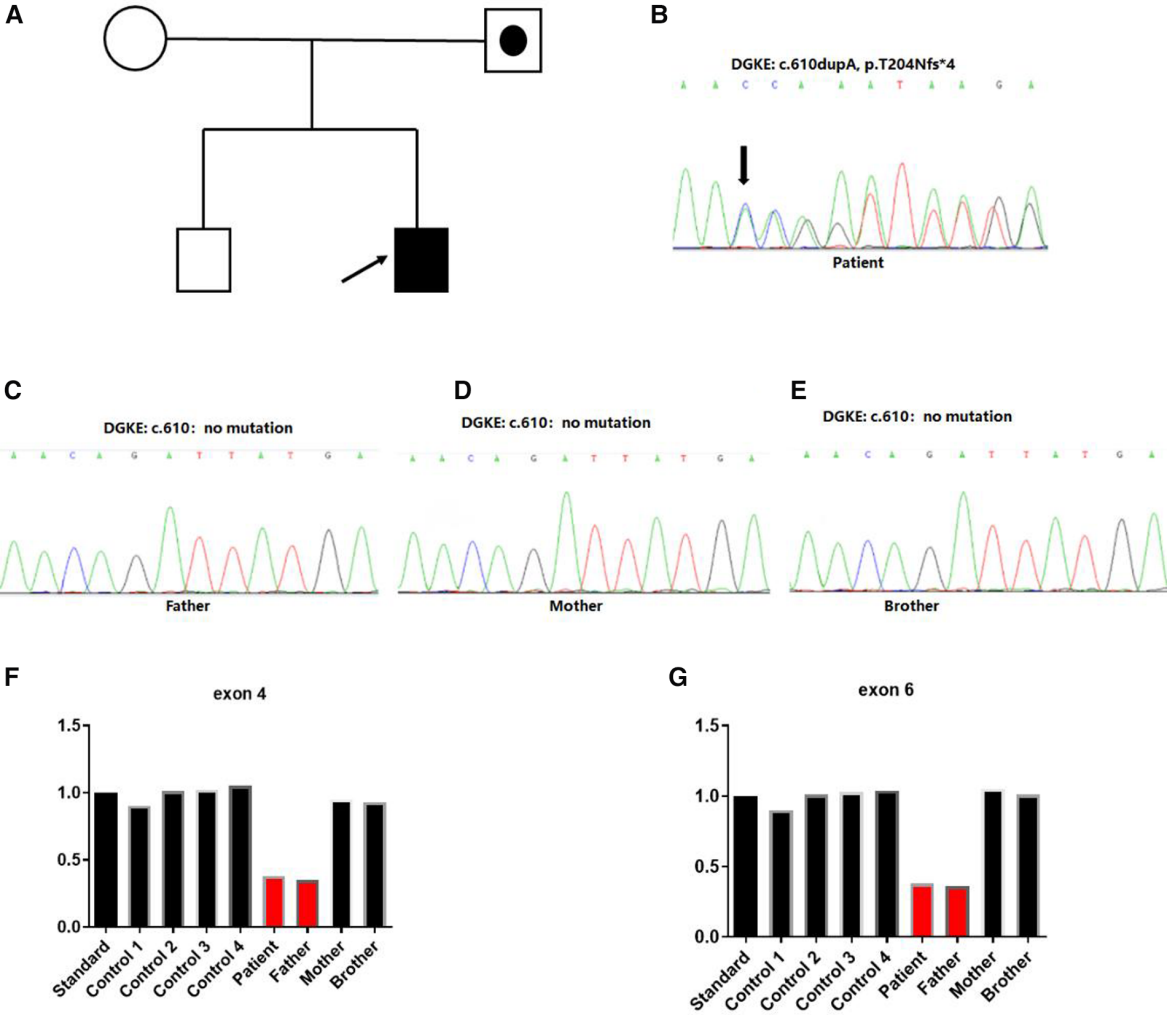
Identification of heterozygous *DGKE* variants in a family with atypical hemolytic uremic syndrome. (**A**) Family history. Index case developed first symptoms of aHUS at 7 months. Squares represent males, and circles represent females. White symbols represent unaffected individuals, white symbols combined with black spots represent individuals with heterozygous deletion variation in the region of exon 4–6 of the *DGKE* gene. Filled black symbols represent affected individuals with the insert variant of c.610dup, p.Thr204Asnfs*4 and heterozygous deletion variation in the region of exon 4–6 of the *DGKE* gene. (**B–E**) Sanger sequencing chromatograms of variants in the *DGKE* gene from the affected patient and his parents. Capital letters represent the exon sequences. The affected individual harbored a compound heterozygous variant of the *DGKE* gene for the c.610dupA; his unaffected parents and brother showed no variant. The arrow indicated the presence of insertional variants. (**F,G**) Heterozygous deletion variant was identified in the 4–6 exon region of the gene.

We obtained 2 ml of peripheral blood from the patient, his parents and older brother respectively. Whole exome sequencing was performed on the patient, his parents and brother. Sanger sequencing was used to verify the mutation site. CNVnator software was used for CNV analysis, and QPCR was used for family analysis. Two heterozygous variations in the *DGKE* exon region: NM_003647.2, c.610dup, p.Thr204Asnfs*4 and deletion of exons 4–6 were identified. The first variant was classified as *de novo* compared with the results obtained in the conventional in the Sanger sequencing of the parents and the older brother (Figures 3A–E). The second variant was a deletion of *DGKE* exons 4–6, which was also detected in the asymptomatic father, but absent in both the mother and the older brother (Figures 3F,G). The patient in this case had aHUS, consistent with recessive transmission with the 100% penetrance.

## 3. Discussion

We described a 7-month-old boy who presented with aHUS with one novel *DGKE* variant for the first time in the Chinese population. The relatively early disease onset and the heartbreaking outcomes reflects the complexity of *DGKE*-aHUS as well as the intractability of treatment.

*DGKE* nephropathy is a rare disease, with clinical features of aHUS, nephrotic syndrome or both, and with aHUS as the most common. The United Kingdom National aHUS Service studies reported that the incidence of *DGKE*-aHUS is 0.009 per million per year (28). Recessive loss-of-function variants in *DGKE* play an essential role in the development of aHUS. In 2013, for the first time, Lemaire M et al. (2) reported that aHUS patients carried the recessive variant of the *DGKE* gene. The *DGKE* (OMIM: 601440) gene is located on chromosome 17q22 and encodes diacylglycerol kinase epsilon (*DGKE*). *DGKE* expression in human endothelial cells and platelets, including the endothelium of glomerular capillaries and podocytes in normal human kidneys, is an important intracellular enzyme (2). *DGKE* preferentially phosphorylates arachidonic acid-containing diacylglycerol (AADAG) to the corresponding phosphatidic acid (PA) and then terminates AADAG signaling and prothrombotic activity. In contrast, AADAGs activate protein kinase C (PKC), which increases the production of various prothrombotic factors in endothelial cells and leads to hemolysis and thrombus formation. In addition, a recent *in vitro* study revealed that the absence of *DGKE* in the endothelium was associated with the production of prostaglandin E2 (PGE2), which controls endothelial activation and the thrombogenic state and results in abnormalities of the glomerular filtration barrier (5, 6, 29). It is therefore plausible that the recessive loss-of-function variants in the *DGKE* gene lead to a prothrombotic state and present with TMA, regardless of clinical or histopathologic findings (27). Elevated AADAG levels can also induce the activation of podocyte calcium cation channels, leading to actin cytoskeletal rearrangements (30–32). A variety of variations in the *DGKE* gene were recorded that result in loss of *DGKE* function. Brocklebank V and his colleagues (20) reported the most common pathogenic genotype was c.966G>A p.(Trp322*). In our case, two *DGKE* heterozygous variants, NM_003647.2, c.610dup, p.Thr204Asnfs*4 and the deletion of exons 4–6 were identified in a Chinese patient with aHUS, even though the first genotype was previously reported (27, 33). To the best of our knowledge, the exon 4–6 deletion is a novel *DGKE* variant, and may be disease-causing.

We know that the diagnosis of *DGKE*-aHUS is made based on a combination of clinical symptoms, laboratory results, genetic analysis, and/or renal biopsy. Renal involvement occurs in all individuals and is mainly present as proteinuria and high serum creatinine levels, with or without hematuria (1, 2, 27). Proteinuria is a distinctive feature of *DGKE*-aHUS as the first symptom in some patients and is maintained for a long time even after the resolution of HUS relapses (27). As reported in a previous study (27), proteinuria was present in all patients at disease onset, and some patients presented with complement activation. An interplay between *DGKE* and complement systems has been suggested by Sanchez CD and his colleagues (7). The finding that podocyte dysfunction with nephrotic-range proteinuria is a complication of this form of *DGKE*-aHUS predisposes patients to the development of TMA. Some patients with combined variants in the *DGKE* gene and the complement system have been identified, which possibly explains the spectrum of clinical manifestations (9, 11, 25). In addition, the loss of various regulators through the leaked kidney and hepatic synthesis of procoagulation factors results in an imbalance of coagulation regulators, which also contribute to the development of TMA, consistent with the pathogenesis of nephrotic syndrome with hypercoagulability. Our case report described a baby presenting with nephritis-type nephrotic syndrome without thrombocytopenia early in the illness. At the time of admission, he had edema, hematuria and massive proteinuria. Other presentations of TMA developed, similar to oliguria and hypertension, along with condition progression. Deng H et al. reported an 8.3-year-old boy with intermittent proteinuria of ∼6 years’ duration who was diagnosed with primary nephrotic syndrome at onset but had episodes of TMA 4 years after diagnosis (15). Bezdicka M and his colleagues observed a baby girl who presented at the age of 8 months with steroid-resistant nephrotic syndrome and later developed thrombocytopenia with progression of AKI after admission (8). Together, these cases show that aHUS can occur after other glomerular diseases and should be considered when thrombocytopenia and hemolytic anemia occur in a patient with nephrotic syndrome. As reported in most of the patients with *DGKE*-aHUS in the literature (10, 11, 14, 19, 22, 24, 25, 26), our patient with *DGKE* variations was diagnosed in the first year of his life. At the time of diagnosis, he had edema, oliguria, petechiae, soy sauce-colored urine, and hypertension. He received PI, PE, CRRT and HD for 6 weeks intermittently, and eventually died with intracranial hemorrhage and multiorgan dysfunction on the 8th week of his hospital stay.

Atypical HUS is a TMA which primarily targets the kidneys, and extrarenal features may occur in the acute phase. The most frequent complication was reported to occur in the central nervous system (CNS) and can occur in 8%–48% of cases (34). The next common extrarenal manifestations of aHUS were respiratory, cardiac and gastrointestinal in nature, as well as integumentary and eye-associated (34, 35, 36). Extrarenal involvement in aHUS was associated with patient prognosis (34, 36). We observed that diarrhea was one of the most common gastrointestinal system complications in the case. While gastrointestinal system involvement is classically associated with the clinical prodrome of aHUS, improvement in gastrointestinal manifestations has been reported in patients with *DGKE*-aHUS after treatment with complement-targeting therapies (9, 13, 16, 26). The direct causal relationship of gastrointestinal manifestations and *DGKE*-aHUS cannot be established from the infrequent case reports at the moment. Additional studies to identify pathophysiological mechanisms are necessary to determine the relationship of *DGKE* variants and gastrointestinal symptoms.

Current therapy for *DGKE*-aHUS is still based on the treatment protocol of aHUS advocated by the European Pediatric Study Group in 2009, including symptomatic treatment, nephroprotective measures, specific treatment of complement-targeting therapies, and kidney transplantation (37). Symptomatic treatments and nephroprotective measures for aHUS are important, which maintain basic life requirements, including strict control of blood pressure, transfusions of red blood cells, anticoagulation, energy support, blockade of the renin–angiotensin–aldosterone system, and metabolic control (37). Early (within 24 h) and intensive PE/PI during the first month of diagnosis are typically the initial treatments for most patients with aHUS, which were beneficial by supplying normal complement proteins, removing mutant proteins or autoantibodies, and preventing podocyte damage (38). The recent study demonstrated that some patients with *DGKE* variants also seemed to respond well to plasma therapy, both PE and PI, whereas they had no benefit for preventing relapse (39). In the same way, plasma therapy did not prevent kidney progression or improve hypertension control in our patient with *DGKE* variants. The efficacy of plasma therapy for *DGKE*-aHUS needs to be studied in a larger number of patients. Eculizumab (ECU), a monoclonal humanized anti-C5 antibody that acts on the terminus of complement activation, prevents C5 cleavage, thus blocking the membrane attack complex MAC and improving abnormal complement regulation. Although the efficacy and safety of ECU have been confirmed for HUS patients (40, 41), it was inconsistent with the benefits of standard complement targeting for *DGKE*-aHUS. Nevertheless, *DGKE*-aHUS patients showed evidence of a response to complete ECU therapy, which may be attributed to the presence of complement activation with or without complement genetic variants (16, 17, 21, 23). Although such case reports are infrequent, a detailed complement analysis evaluation for *DGKE*-mediated aHUS should be considered in patients with specific treatment with comple ment-targeting therapies, including plasma and ECU therapy. No evidence of complement activation was observed in some patients, and several relapses occurred after complement-inhibiting therapy. These findings indicate an important role of complement activation in response to therapies in aHUS patients with *DGKE* variants. We agree with the fact that the cause-effect relationship between ECU and the remission of *DGKE*-aHUS cannot be absolutely certain and that ECU should be safely withdrawn in these individuals without markers suggestive of complement activation to avoid adverse effects, including severe infections. A randomized controlled study of patients with *DGKE* variants might be beneficial for drawing an association between the treatment of complement-targeting therapies and *DGKE*-mediated aHUS.

A subset of patients with autoantibodies for *DGKE*-aHUS are also treated with corticosteroids and immunosuppressants (12). Notably, other immunosuppressive therapies, such as rituximab (a humanized monoclonal antibody against B-cell surface antigen CD20), have been prescribed for aHUS patients with *DGKE* variants to eliminate antibody formation and sustain long-term remission (8, 18). There was no benefit from the treatment of rituximab combined with corticosteroids and cyclosporin demonstrated in a 4-month-old baby with a *DGKE* variant and complement activation, while the benefit was uncertain—with clinical improvement but CKD stage 3 and persistent proteinuria—in one patient with an isolated *DGKE* variant. The published literature (20, 27) reports the outcomes in 6 kidney transplant recipients, and the risk of posttransplant recurrence in patients with *DGKE* variants is zero. In summary, the optimal management strategies for patients with aHUS and *DGKE* variants have not yet been defined. Additional studies to replicate these findings are necessary to determine whether ECU and/or rituximab are beneficial for individuals with *DGKE* variants, particularly for children with combined *DGKE* gene variants and complement activation or positive antibodies.

Our case report leaves several limitations. First, due to the limited implementation of some laboratory projects, the degradation products of fibrinogen, the laboratory examination for the activation of alternate complement pathways could not be obtained for timely inspection. Second, our study involved proband diagnosis till 3 generations. The patient's grandmother died of some hematological problems before he was born. The cause of her death is unknown and specific clinical data was missing. The LOD score was difficult to obtain. Third, this study included the sample data for study remained incomplete. A high-quality research to clarify the clinical features and management of aHUS patients with *DGKE* gene variants is needed in the forthcoming period.

## 4. Conclusion

In conclusion, our study further expands the spectrum of the sequence variants reported in the *DGKE* gene and reports the first aHUS patient with a *DGKE* variant within China. Our case instructively highlights the need to suspect noncomplement culprits such as *DGKE*-aHUS with thrombocytopenia and hemolytic anemia occurring in young patients with nephrotic syndrome. Moreover, the present literature suggests that aHUS with *DGKE* variants can occur at any age but typically manifests in the first year of life and has a poor prognosis with a higher rate of mortality characterized by early-onset relapsing HUS with rapidly worsening renal function, chronic proteinuria and long-term progression to CKD (27). There is no specific treatment for *DGKE*-aHUS. There have an uncertain benefit of plasma therapy for *DGKE*-aHUS patients. Complement targeting and immunoregulatory therapies present some benefit for *DGKE*-aHUS with complement activation and autoantibodies during overt TMA presentation, but they do not prevent kidney progression and TMA relapse. It is critical to identify genetic defects, and evaluating detailed laboratory parameters (complement analysis and autoantibodies) for aHUS can help confirm the diagnosis of noncomplement-mediated disease and potentially determine treatment therapies and duration. High-quality prospective studies to clarify the association between the treatment of complement-targeting therapies and *DGKE*-mediated aHUS are needed in the future.
